# Germline and Embryonic Mechanisms in the Epigenetic Inheritance of Neurodevelopmental and Cognitive Traits in Mammals

**DOI:** 10.3390/biom16050669

**Published:** 2026-05-01

**Authors:** Mehmet Kizilaslan, Zeynep Kizilaslan, Hasan Khatib

**Affiliations:** Department of Animal and Dairy Sciences, University of Wisconsin-Madison, Madison, WI 53706, USA; kizilaslan@wisc.edu (M.K.); zkizilaslan@wisc.edu (Z.K.)

**Keywords:** germline, epimutation, embryo, intergenerational, transgenerational, epigenetic inheritance, neurodevelopment, nervous system, cognitive, mammals

## Abstract

Epigenetic mechanisms profoundly regulate gene expression, developmental trajectories, and phenotypic variation, extending biological influence beyond DNA sequence alone. A growing body of evidence suggests that environmental exposures, including pollutants, drugs, stress, and diet, can induce germline and early embryonic epimutations that alter developmental programs with lasting consequences for neurodevelopmental and cognitive outcomes. However, the fields most relevant to these processes have largely developed independently. These include germline epigenetics, early embryonic patterning, neurodevelopment and cognitive regulation, and intergenerational or transgenerational inheritance. Each field has its own conceptual frameworks and mechanistic models. This fragmentation obscures the biological reality that these systems are tightly interconnected: environmentally induced epigenetic perturbations in gametes can reshape the epigenetic landscape of the early embryo, influence lineage allocation during gastrulation, and ultimately modify the molecular architecture of the developing central nervous system. A systems–biology perspective capable of linking germline epimutations and early embryonic epigenetic instability to later neurodevelopmental and cognitive phenotypes and their potential inheritance is therefore required. This review synthesizes current evidence across these traditionally isolated domains and proposes a coherent mechanistic framework linking germ cell epimutations and early embryonic epigenetic instability to the emergence of neurodevelopmental and cognitive phenotypes. By bridging these conceptual gaps, we aim to establish a cohesive foundation for understanding how early epigenetic disruptions generate long-lasting and in some cases heritable effects on brain development and cognitive function.

## 1. Introduction

Environmental epigenetics play a central role in the regulation of gene expression patterns, phenotypic variation, and the inheritance of traits beyond DNA sequence differences [1,2]. Factors such as chemical pollutants, drugs, heat, stress, and diet, as well as other environmental inputs, can induce epigenetic modifications that can influence developmental trajectories, disease susceptibility, and metabolism, with long-lasting effects that may be inherited across many subsequent generations [3,4,5]. Following gamete fertilization, precursor cells commit to their fate in a stepwise differentiation process that begins in a single cell, influenced by multiple inputs (e.g., environmental stimuli and genetic variation) and is driven by accompanying epigenetic modifications that reinforce commitment [6,7]. Such epigenetic modifications, once established, are stable and heritable through mitosis, hence propagated across tissues, organs, and systems over many cell divisions, affecting transcription profiles [6,8]. Epimutations—epigenetic alterations in gene activity that occur without changes in DNA sequence—arising during critical developmental windows, particularly gametogenesis, preimplantation, and early embryonic stages, are expected to alter offspring development and physiology. These fetal programming effects may persist throughout postnatal life, consistent with the Developmental Origins of Health and Disease (DOHaD) hypothesis [9,10]. Fetal programming refers to the process by which these early exposures shape long-term biological set points, often through persistent epigenetic remodeling. Beyond that, altered epigenetic states and phenotypic outcomes can be inherited through the germline to subsequent generations, leading to a cascade of exposed and unexposed generations with affected phenotypes. Environmental stimuli that alter the germline and embryonic epigenetic landscape lead to intergenerational (IEI) or transgenerational epigenetic inheritance (TEI). Although parental exposures directly influence intergenerational epigenetic effects, transgenerational inheritance reflects changes that persist beyond direct exposure, implying stable epigenetic reprogramming within the germline with accompanying traits [4,11].

**Figure 1:**
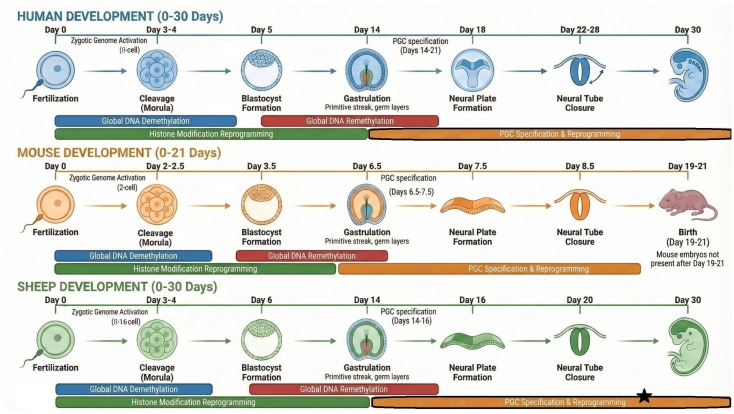
Early developmental time scale of three different mammalian organisms: human, mouse, and sheep. The figure demonstrates early embryonic development of the organisms (approximately a 30-day period). In mice, all these important developmental stages are packed into the first ~10 days, as gestation lasts ~19–21 days. * Although the exact timing of PGC epigenetic reprogramming in sheep is not known, it is expected to follow patterns similar to humans, based on studies of PGC development in sheep [28,29,30].

Neurodevelopmental and cognitive phenotypes are remarkably sensitive to early epigenetic perturbations for several reasons. The central nervous system (CNS) initiates specification earliest among other organ systems with neural induction, partially coinciding with the primordial germ cell (PGC) formation and transportation period, and serves as the locational framework for the subsequent organogenesis [12,13]. Simultaneously with the gastrulation and primitive streak formation, neural plate induction occurs, followed by neurulation, which includes neural tube formation and closure (Figure 1). This early developmental sequence is crucial for establishing the body’s template axial symmetry of all three directions (anterior–posterior, dorsal–ventral, and left–right) and cues for positioning the internal organs. These events require tightly regulated biological processes, including cell proliferation, migration, adhesion, ciliary function, and complex signaling, as well as biomechanical activities such as elevation, folding, bending, and coordinated cell movements [14]. Importantly, this entire developmental window coincides with a period of profound epigenomic plasticity, during which DNA methylation patterns, histone landscapes, and chromatin accessibility are being extensively rewritten (Figure 1). The developmental timeline in Figure 1 highlights this temporal overlap between epigenetic events in early embryos and the onset of neural induction, illustrating a critical window in which persistent germline epimutations may disproportionately influence lineage specification. By aligning these processes on a shared temporal axis, the figure emphasizes how environmentally induced alterations in germline or early embryonic epigenetic marks can intersect with early neurodevelopmental decisions. Since many other organ systems develop downstream of these early patterning events, disruptions to neural lineage specification can propagate broadly across embryonic development and profoundly influence postnatal life. Moreover, neuronal differentiation, synaptogenesis, and circuit assembly rely heavily on fine-tuned epigenetic regulation, making the brain particularly vulnerable to even subtle alterations in early epigenomic programming [15]. Finally, CNS is also among the last systems to complete its development in certain regions and neural circuits, which spans an extended period of postnatal life in mammals [16].

The formation of the nervous system is organized by precisely timed genetic and epigenetic regulatory programs that interact with the prenatal environment [16]. Perturbations during these early windows can divert developmental pathways, resulting in absent, malformed, or improperly connected neuronal circuits. Epigenetic mechanisms play a central role in neurodevelopmental and cognitive traits, including learning and memory, as well as disorders such as Fragile X and Rett syndromes, Huntington’s disease, schizophrenia, bipolar disorder, and neural tube defects. These mechanisms are highly responsive to environmental inputs, including folate and one-carbon metabolism, retinoic acid, medications, chemical exposures, physical activity, and stress [17,18,19,20,21,22]. Understanding the molecular components and environmental conditions that cause or result in epigenetic changes may offer unique opportunities to develop novel interventions and therapies for a range of neurological and psychiatric conditions. Yet the action mechanisms and inheritance underlying these complex interactions remain poorly understood.

Most epigenetic studies linking environmental exposures to neurodevelopmental and cognitive outcomes have relied on rodents, despite growing evidence that mice and rats do not fully model human neurodevelopment. Key discrepancies, including major physiological and anatomical differences, compressed embryonic patterning, shorter gestation, and species-specific inheritance patterns for disorders such as congenital neural tube defects, limit their translational relevance [23,24,25]. These constraints have renewed interest in large-animal systems that more closely mirror human developmental biology. Sheep are particularly valuable models because of their physiological similarity to humans, their suitability for studying naturally occurring neurological diseases, and their established ‘gold standard’ use in fetal surgical models of neural tube defects [23,26,27]. Alongside these attributes, sheep provide a uniquely advantageous large-animal model. They possess a human-like gyrencephalic brain absent in rodents, a practical ~150-day gestation that enables faster multigenerational epigenetic studies than cattle (~280 days), tighter dietary, environmental, and genetic control than canine models, and a more human-like litter size compared to pigs (~8–14 offspring). Collectively, these features make sheep superior to other large-animal models (e.g., cattle, dogs, pigs) for investigating neurodevelopmental and epigenetic mechanisms. Our recent work further underscores the relevance of sheep as a model. A moderate F0 methionine supplementation to F0 rams was found to induce epigenetic inheritance across five generations of sheep, with sperm methylation changes enriched for neurodevelopmental pathways and associated phenotypes in growth and fertility [3]. Importantly, Figure 1 also illustrates how the timing of early embryonic events, including epigenetic reprogramming, germ layer formation, and neural induction, aligns far more closely between sheep and humans than between rodents and humans. This temporal concordance during the first ~30 days of development strengthens the rationale for using sheep to study environmentally induced germline epimutations and their potential influence on early neurodevelopmental trajectories. However, research on the epigenetic inheritance of neurodevelopmental and cognitive traits in sheep remains in its early stages.

Despite broad recognition of how environmental exposures interact with epigenetic regulation to influence neurodevelopment and cognition, the field of neurodevelopmental epigenetic inheritance remains conceptually fragmented and compartmentalized. A unified systems–biology framework that connects germline epigenetic perturbations to early embryonic patterning and later neurodevelopmental trajectories remains lacking. Germline epigenetics, early embryonic patterning, neurodevelopment, and epigenetic inheritance remain largely isolated domains, each with distinct conceptual and mechanistic models. This separation obscures the fact that these processes are tightly interconnected. Environmental signals encountered by gametes and embryos shape the epigenetic landscape of early development, influence lineage patterning during gastrulation, and ultimately alter the molecular architecture of the developing central nervous system, including the spinal cord and brain (Figure 1). A cohesive framework linking gametes to brain epigenetics is therefore essential for understanding how early exposures produce long-lasting and sometimes heritable neurological outcomes. Therefore, the aim of this review is to establish a coherent mechanistic framework linking germ cell epimutations and early embryonic epigenetic instability to the emergence of neurodevelopmental and cognitive phenotypes and their epigenetic inheritance.

## 2. Germline Epimutations: Mediators Between the Environment and Neurodevelopmental and Cognitive Traits

Germline epimutations serve as a critical mediator between parental environments, molecular perturbations during gametogenesis, and the early embryonic programs that shape neurodevelopmental and cognitive outcomes. Their origins, molecular carriers, and mechanisms of persistence collectively define the potential for intergenerational and transgenerational inheritance associated with the etiology of neurodevelopmental and cognitive traits.

### 2.1. External Sources of Germline Epimutations

A diverse set of biological and environmental factors is associated with the induction of stable epigenetic alterations in the germline. Various environmental stimuli, including diet, chemical exposure, smoking, aging, endocrine disruptors, trauma, heat stress, and chronic psychological stress, have repeatedly been shown to modify epigenetic profiles in sperm [3,31,32,33,34,35,36,37] and in oocytes [38,39]. Many metabolic signaling regulators, genes of oxidative stress pathways, chromatin state regulators, RNA modification enzymes, one-carbon metabolism, histone and DNA methylation machinery seem to be heavily affected by the altered epigenetic status in response to environmental stimuli. Mechanistically, most of these influences manifest as DNA methylation errors, aberrant histone retention, and small RNAs, each of which has been documented in major human and animal studies examining environmentally induced germline epimutations. Notably, several of these studies report enrichment of epimutations at loci implicated in neuronal differentiation, synaptic function, and risk for neurodevelopmental disorders.

### 2.2. Molecular Mechanisms of Epigenetic Inheritance in Gametes

**Figure 2:**
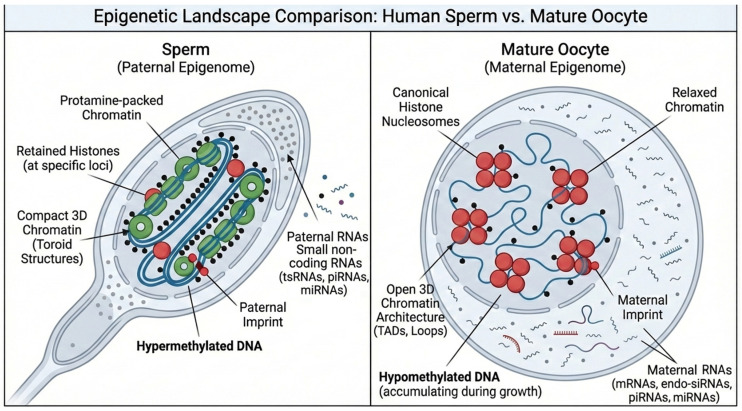
Generalized representation of key epigenetic modifications in germline cells, illustrating common patterns of DNA methylation, histone marks, and small RNAs. On the left, the epigenomic landscape of sperm cells shows a tightly packed “ready-for-delivery” paternal genome, in which most histones are replaced by protamines, an aspect that will be reversed following fertilization. On the right, the epigenomic state of a mature oocyte is shown, with a loose open 3D chromatin structure of the maternal epigenome, where histones are preserved and are mostly populated by mRNAs, miRNAs, endo-siRNAs, and piRNAs.

Multiple molecules have been shown to carry environmentally induced epigenetic information in mammalian gametes. Although sperm and oocytes differ remarkably in chromatin architecture, transcriptional activity, and cytoplasmic content, both germ cells harbor molecular features that can encode, retain, and transmit environmentally responsive epigenetic states to the early embryo. Figure 2 illustrates these complementary epigenomic landscapes, contrasting the highly compact, protamine-rich paternal genome with the open histone-retaining maternal chromatin architecture. This visual comparison highlights how the two gametes contribute distinct but synergistic epigenetic information to the early embryo. DNA methylation and hydroxymethylation, histone modifications (including methylation, acetylation, phosphorylation), small non-coding RNAs (including tsRNAs, piRNAs, and miRNAs), as well as chromatin 3D structure are now recognized as potential carriers of epigenetic information in gametes, and they serve as mediators of epigenetic inheritance across early embryos [6,40,41,42].

Mature sperm have a tightly packed genome, where >85% of histones are replaced by protamines in mice and humans, leaving only ~1–15% nucleosomes at key development promoters and imprinted loci, which are enriched for H3K4me3, H3K27me3, and H3K9me3, forming regulatory islands that persist in the zygote [43,44,45]. Mature sperm also exhibit very high global CpG methylation (~70–90%), fully established paternal imprints, and a small-RNA content dominated by tsRNAs, miRNAs, and piRNAs, which are further remodeled during epididymal transit [46,47,48]. In contrast, mature oocytes retain a full histone complement, display lower global DNA methylation (~40%), and establish maternal imprints through transcription-coupled DNMT3A/3L activity [48,49]. Oocytes also feature broad non-canonical H3K4me3 domains across promoters and distal regulatory regions [50]. They additionally carry H3K27me3-based imprinting at non-methylated loci to prevent chromatin accessibility [51]. The small-RNA content of oocytes is mostly dominated by maternal miRNAs, piRNAs, and endo-siRNAs that regulate meiotic progression and maternal mRNA clearance [52]. On the other hand, recent studies suggest that mRNA methylation, particularly m6A modifications, plays a role in sperm and can be inherited across embryos [53,54]. Together, these complementary epigenetic architectures, visually summarized in Figure 2, reflect the sperm’s role in delivering a compact methylation-rich paternal genome and the oocyte’s role in providing a transcriptionally active histone-rich maternal genome, both of which are essential for early embryonic reprogramming. These epigenetic information carriers form a multimodal system through which germline epimutations can influence the earliest stages of lineage specification, including neuroectodermal fate.

### 2.3. Inheritance for Germline Epimutations in Early Embryos

Multiple epigenetic mechanisms allow environmentally induced epimutations to persist through the extensive epigenetic reprogramming (see details in Section 3) following fertilization and during PGC formation at early embryonic gastrulation, which is immediately followed by neural tube formation. These mechanisms perform the intricate task of inheritance of ‘acquired’ epigenetic marks through either direct replication or indirect reconstruction of parental states driven by other epigenetic markers [42], enabling the embryo to inherit molecular ‘memories’ of parental environments. In this section, the most well-known mechanisms of environmentally induced epigenetic mark transmission are reviewed.

Certain genomic regions, including imprinted genes, transposable elements (e.g., LINE, SINE), metastable epialleles, and CpG-dense regulatory loci, partially resist global demethylation [55,56,57]. These ‘escapee loci’ can preserve parental epigenetic states that subsequently influence early embryonic gene regulation, including genes involved in neural patterning and synaptic development. Almost 90% of the escapee loci are transposable elements [57]. Strikingly, a substantial proportion of escapee loci in CpG-rich regions map to genes regulating neurological and neurodevelopmental processes, emphasizing their disproportionate involvement in pathways critical for CNS formation and function [3,55,56].

Sperm- and oocyte-packed small RNAs can persist and affect early embryonic transcription, regulate transposable elements, recruit chromatin modifiers and remodel 3D chromatin structure, and stimulate direct locus-specific methylation changes (Figure 2) [42,58]. These actions create a functional intervention that alters parental exposures, which can affect developmental pathways, including those governing neuronal proliferation, axon guidance, and cortical maturation. Key classes of small RNAs involved in epigenetic inheritance include tsRNAs, piRNAs, and miRNAs in sperm, whereas oocytes predominantly carry miRNAs, piRNAs, and especially endo-siRNAs [59]. These are shown to be altered by environmental factors such as diet, stress, and metabolic states; regulate translation and early zygotic transcription; guide de novo DNA methylation at transposons and some gene promoters; guide chromatin modifier enzymes to specific loci (e.g., DNMTs, TETs, HDACs, and histone methyltransferases); and regulate mRNA clearance and early lineage-specification pathways [42,60,61].

Histone retention is another mechanism of transmission for environmentally induced epigenetic marks. A small proportion of sperm chromatin (~1–15%) retains histone-based nucleosomes that are non-randomly enriched at promoters of developmental regulators, especially for the nervous system and embryonic development, including *HOX* gene clusters, parentally imprinted domains, neurogenic enhancers (e.g., LINEs), and, in some cases fertility related genes [43,44,62,63]. These retained histones frequently carry regulatory marks such as H3K4me3, H3K4me2, H3K27me3, and H3K9me3, which persist through fertilization and can influence early embryonic chromatin organization. In parallel, the maternal genome preserves most of its histone landscape, including broad H3K4me3 and H3K27me3 domains in the mature oocyte [51]. Brought together by fertilization, these paternal and maternal histone reservoirs can determine early embryonic chromatin states, regulate zygotic genome activation, and affect lineage allocation, including early neural trajectories [44,51].

Environmental exposure influences not only DNA methylation or histone modifications but also the higher-order 3D architecture that contributes to chromatin shape memory [42]. Chromatin structures such as CTCF/cohesin-delimited topologically associating domains (TADs), lamina-associated domains that maintain repressive nuclear positioning, and enhancer–promoter loops that encode long-range regulatory relationships are partially inherited or reconstructed from parental markers [64,65]. Gamete-delivered architectural proteins and retained chromatin structures bias the early embryonic rebuilding of 3D regulatory topology, affecting lineage-specific transcriptional programs, including those essential for CNS and especially brain development [66,67].

Epigenetic inheritance studies are extremely scarce on metabolic memory, although parental metabolic signatures determine the germ-cell metabolite pools, including the SAM/SAH ratio, α-ketoglutarate, and acetyl-CoA, each of which directly regulates the activity of DNMTs, TET dioxygenases, and histone acetyltransferases following fertilization. Metabolic perturbations during gametogenesis can imprint persistent “metabolic signatures” on chromatin, modifying DNA methylation, histone methylation dynamics, and histone acetylation, thereby biasing early embryonic epigenetic programming and pathways such as neuronal energy metabolism and neurogenesis. One-carbon metabolism, particularly the SAM/SAH ratio, is closely linked to epigenetic inheritance of neurodevelopmental disorders, including NTDs, which arise from failed neural tube closure during early embryogenesis (~week 3 of human gestation; Figure 1), following epigenetic reprogramming associated with primordial germ cell formation.

### 2.4. Germline Epimutations in Neurodevelopmental and Cognitive Traits

Across human cohorts and animal models, environmentally induced germline epimutations are consistently observed in regulatory regions, including CpG-dense elements, imprinted loci, and transposon-derived enhancers. Although not explicitly, several exposure models report affected loci within neurodevelopmental and signaling pathways, consistent with the known epigenetic sensitivity of neuronal enhancers, like transposons [3,55,68]. Epimutations at neurodevelopmental loci, including regulators of neuronal differentiation, axon guidance, cortical layering, and neurotransmission, have been identified in sperm from diet-exposed fathers, in metabolically or environmentally stressed oocytes, and in tissues of offspring with altered neurobehavioral phenotypes. These findings support a model in which environmentally induced germline epimutations modify early embryonic epigenetic stability, shaping neurodevelopmental pathways prior to neural tissue formation. Through escape from reprogramming, reconstruction by germline-derived factors, and small-RNA-mediated modulation of early transcription, these epimutations can shape the molecular architecture underlying neurodevelopmental and cognitive traits across generations. Although transgenerational epimutation inheritance is discussed in later sections, we here highlight selected studies that illustrate the pathway from environmental stimuli to germline epimutations, their transmission during early embryogenesis, and the consequent effects on neurodevelopmental pathways.

For example, paternal environmental exposures, including low-protein or high-fat diets in mice, induce sperm DNA methylation and small RNA alterations at loci enriched for growth factor and neurodevelopmental regulators, including *IGF2*, *H19*, and genes within Wnt signaling, axon-guidance, and folate metabolism pathways. These alterations are accompanied by corresponding changes in offspring metabolic and stress pathways relevant to brain function [32,58,69,70]. Similarly, chronic paternal stress in rodents remodel sperm miRNA and tsRNA profiles, targeting genes involved in glucocorticoid signaling and synaptic plasticity, leading to altered hypothalamic–pituitary–adrenal axis function and anxiety-like behaviors in the progeny [71]. In large-animal models, a paternal methionine-rich diet in sheep induces stable differentially methylated loci in sperm at genes enriched for neurodevelopmental pathways, including those involved in neuronal differentiation and cortical patterning. These changes correlate with inherited developmental phenotypes [3,72]. On the maternal side, environmental insults such as obesity, undernutrition, endocrine disruptors, and heat stress have been shown to reprogram oocyte DNA methylation and histone marks at imprinted loci, chromatin regulators, and planar cell polarity genes, with downstream effects on early lineage allocation, nervous system development and brain growth trajectories [38,73]. Consistent with animal studies, human sperm methylome analyses in exposed cohorts (e.g., to smoking or endocrine-disrupting chemicals) reveal environmentally sensitive differentially methylated regions. These regions overlap genes involved in synapse organization, neuronal differentiation, neurodevelopmental disorder risk, and fertility. These findings suggest that a subset of environmentally induced germline epimutations may directly affect neurodevelopmental gene networks while influencing reproductive outcomes in subsequent generations [36,74,75].

## 3. Sources of Epigenetic Instability in Early Embryonic Development: A Critical Bottleneck

Fusion of the mature sperm and oocyte at fertilization unites two genomes and cytosolic environments extensively shaped by environmental exposures, metabolic states, and life-history events. Upon zygote formation, however, these non-identical parental genomes and epigenomes enter one of the most destabilizing phases of the life cycle. During early embryogenesis, epigenetic marks are subjected to diverse intrinsic and extrinsic influences that challenge their stability, determining whether they are maintained or remodeled across developmental stages and generations. This period represents a window of maximal epigenomic plasticity and vulnerability, during which persistent lineage-specific differences can arise. Although epigenetic reprogramming provides the zygote with a developmental “reset”, enabling differentiation, compartmentalization, and organismal formation from highly specialized gametes [76,77], the error-prone nature of this process creates a phase of exceptional susceptibility. This narrow period encompasses implantation, gastrulation, PGCs, the primitive streak, body axis establishment, initiation of neural induction, neural plate formation, and the morphogenetic events of neural tube closure (Figure 1). Each of these processes relies on precisely timed epigenetic remodeling at critical developmental loci, including *WNT*, *NODAL*, *BMP*, *FGF*, and neural competence genes [14]. Perturbations to the stability of inherited or newly established epigenetic states during this interval can therefore compromise all these processes of neural tube morphogenesis, increasing susceptibility to neurodevelopmental disorders and structural defects. Although PGCs and the CNS initiate specification earlier than other organ systems during this period, epigenetic changes at this stage can also have cascading effects on other developmental trajectories [10,12,13,78]. The early embryo develops with non-identical parental epigenomes, and these inherited marks contribute to initial variability. Such intergenerational asymmetries shape the epigenetic landscape even before reprogramming begins. In this section, we briefly review the major sources of epigenetic variability and instability to highlight mechanisms that can disrupt the inheritance of environmentally induced germline epimutations.

### 3.1. Global Epigenetic Reprogramming and Zygotic Genome Activation

Global epigenetic reprogramming and zygotic genome activation (ZGA) represent two major sources of epigenetic change in early embryogenesis. Following fertilization, parental epigenomes undergo rapid, global, and highly asymmetric reprogramming, including waves of DNA demethylation, chromatin remodeling, histone replacement, small RNA turnover, and 3D genome reorganization. These processes are essential for establishing totipotency but simultaneously create a bottleneck for the fidelity of inherited epigenetic information [42,77,79]. This reprogramming process starts following fertilization and results in the formation of a totipotent zygote [80,81]. Zygotic genome transcription initiates around the 2-cell and 8-cell stages in mice and humans, respectively, encompassing this period of reprogramming [79]. Different epigenetic marks complete reprogramming at distinct time points, exhibiting pronounced asymmetry between sperm and oocyte epigenomes. Although the timing of zygotic genome activation varies across species, reprogramming is largely complete by the blastocyst stage in most mammals [79]. Following embryo implantation, PGCs differentiate from epiblast cells and undergo a second wave of epigenetic reprogramming, erasing parental epigenetic signatures and imprinting markers [82]. This reprogramming represents an extended period of epigenetic erasure, beginning during PGC migration toward the genital ridge and passing near the primitive streak. In humans, complete PGC colonization of the genital ridges occurs around 5–6 weeks post-fertilization, with extensive demethylation and reprogramming largely completed by 7–9 weeks [57,83,84]. During PGC differentiation, migration, and concurrent epigenetic reprogramming, the post-implantation embryo initiates neural induction, with the epiblast forming the primitive streak, embryonic germ layers, neural plate, and completing neural tube closure between weeks 2 and 4 post-fertilization in humans (Figure 1). As germ cells mature, sex-specific epigenetic modifications are established to guide meiosis and germline development.

These two epigenetic reprogramming events constitute a developmental bottleneck in which intrinsic molecular processes, extrinsic environmental conditions, and stochastic fluctuations interact to determine whether germline acquired epimutations are erased, retained, or reconstructed into stable developmental trajectories. Certain classes of epimutations, including imprinted regions, germline-resistant CpG islands, transposable-element derived enhancers, and environmentally responsive small-RNA pathways can partially escape these erasure points and transmit molecular “memories” of parental exposures into the early embryo. Depending on the nature of the inherited signal, these marks may either buffer the embryo against environmental fluctuations or sensitize it to metabolic, nutritional, or endocrine stressors, thereby influencing neurodevelopmental trajectories in adaptive or maladaptive directions. In this context, early embryogenesis represents a period of heightened developmental vulnerability and a window for inter- and transgenerational transmission of acquired traits. This is particularly true for traits affecting neural development, stress responsivity, and behavior, allowing offspring phenotypes to be shaped—positively or negatively—by the ecological and physiological experiences of previous generations.

### 3.2. Other Epigenetic Variability Sources of Early Embryonic Development

Beyond intrinsic reprogramming events, maternal environmental factors during conception and early embryogenesis are major drivers of epigenetic variability. Well-known influences include maternal age, folate and other methyl donor availability, metabolic conditions (such as obesity, diabetes, and insulin resistance), stress, inflammation, exposure to endocrine disruptors and environmental toxicants, and oocyte mitochondrial quality [38,85,86,87]. These factors alter the activity of DNMTs, TETs, histone modifiers, and metabolic cofactors (SAM, α-KG, acetyl-CoA).

During early embryonic development, variability also arises from the dynamics of epigenetic enzymes and from mutations. Key contributors include fluctuations in DNMT3A/3B (de novo methylation), DNMT1 (maintenance methylation), TET enzymes (active demethylation), histone methyltransferases and acetyltransferases, and chromatin remodelers [6,88]. Small shifts in enzyme abundance or activity during rapid cell cycles produce lasting epigenetic differences, stabilizing or destabilizing germline epimutations.

The early embryo undergoes dramatic 3D genome reorganization through TAD resolving and reformation, CTCF/cohesin-regulated looping changes, nuclear lamina repositioning, and chromocenter formation [79,89]. Interestingly, a recent study suggests that a brief pulse of histone deacetylase inhibition can regulate transcription, histone modifications, and genome folding [90]. Moreover, transcriptional deregulation at specific loci and altered genome architecture can partially preserve the perturbed chromatin conformation even after the inhibition is reversed. These transitions influence enhancer–promoter interactions and create variability in chromatin accessibility.

Transposable elements (TEs) are unusually active in early embryos, especially in terms of LINE-1 transcription, ERV activation, and TE-derived enhancers, which are widely known for their role in CNS differentiation [91,92]. The 3D genome reorganization is also known to be highly influenced by TE activity during early embryogenesis [93]. These TE activities reshape chromatin accessibility and methylation patterns, adding an extra layer of regulatory complexity.

As the embryo forms trophectoderm, inner cell mass, epiblast, and embryonic germ layers, signaling pathways generate epigenetic divergence. The Hippo, FGF/ERK, Wnt, BMP, and Nodal signaling pathways recruit chromatin modifiers to establish lineage-specific epigenetic states [94]. Recently, we conducted a multi-omics study to identify molecular drivers of spina bifida, a neural tube defect. Our analysis revealed genes associated with key pathways—particularly Wnt and BMP—as well as histone modifiers and methylation enzymes, with epimutations linked to spina bifida in sheep (unpublished data). The metabolic state of the early embryo directly feeds epigenetic reactions through the SAM/SAH ratio, changing methylation potential, α-ketoglutarate, and Acetyl-CoA abundances, thereby regulating TET activity and histone acetylation. These shifts in metabolic flux at early embryonic period create variability in epigenetic mark regulation [95].

Implantation introduces additional sources of variability in epigenetic marks. Mechanical forces, uterine signaling molecules, epiblast polarization, and the breaking of embryonic symmetry drive post-implantation remodeling while challenging the retention of previously established marks. These cues reshape chromatin landscapes as the embryo transitions to post-implantation development [79,96,97]. Moreover, as the embryo goes through gastrulation, forming ectoderm, mesoderm, and endoderm; lineage-specific enhancers activate, chromatin accessibility diverges, DNA methylation landscapes become cell-type-specific, and signaling gradients (Wnt, Nodal, BMP) reshape epigenetic patterning. This also creates a pattern of variability, and an environment either enforces or removes certain escapee epigenetic marks [98,99,100].

## 4. How Do Early Epigenetic Disruptions Alter Neurodevelopment and Cognition?

Neurodevelopmental and cognitive traits are profoundly shaped by the earliest structural and molecular events of embryogenesis regulated by environmental stimuli through epigenetic variation [101]. Across this window, germline-derived and early embryonic epigenetic processes and metabolic regulation of chromatin compounds act as foundational determinants of core neurodevelopmental phenotypes, cognitive abilities, and later-emerging neuropsychiatric and behavioral traits [102].

Environmental epigenetic perturbations during early embryonic neurodevelopment establish long-lasting developmental biases that increase susceptibility to a broad spectrum of disorders, including autism spectrum disorder (ASD), intellectual disability, schizophrenia, depression, anxiety, epilepsy, social deficits, ADHD, bipolar disorder, microcephaly, and NTDs such as spina bifida and anencephaly [16,103,104,105,106,107]. For instance, early epigenetic perturbations in Wnt signaling during neural induction alter dorsal signaling relevant to ASD, disruptions in Shh/BMP gradients during ventral forebrain patterning bias interneuron development implicated in schizophrenia, and defects in planar cell polarity (PCP) signaling during neural tube closure predispose to NTDs [108,109,110].

These early developmental processes also shape core cognitive domains, including working memory, executive function, processing speed, and language acquisition, through their effects on cortical neurogenesis, radial migration, laminar patterning, and synaptic circuit assembly [16,111]. Such outcomes reflect the combined influence of germline-derived and early embryonic epigenetic mechanisms, DNA methylation reprogramming, histone retention and post-translational modifications, chromatin accessibility, small-RNA inheritance, and metabolic–epigenetic coupling, which regulate lineage allocation and prenatal cortical development. Importantly, these epigenetic systems are highly sensitive to environmental perturbations during the preconception period and early embryogenesis. For example, maternal folate deficiency induces hypomethylation at key developmental genes during neural tube closure, maternal inflammation disrupts fetal cortical lamination and enhancer accessibility relevant to ASD, prenatal alcohol exposure alters chromatin accessibility at forebrain enhancers associated with epilepsy, and paternal stress or diet reshapes sperm small-RNA cargo, influencing offspring attentional control and cortical development [71,112,113]. Together, these findings demonstrate that early embryonic and environmentally responsive epigenetic processes act as foundational determinants of neurodevelopmental phenotypes, cognitive ability, and later-emerging neuropsychiatric and behavioral traits.

## 5. Epigenetic Inheritance of Germ Cell and Embryonic Epimutations Affecting Neurodevelopmental and Cognitive Traits

Epimutations in germ cells and early embryonic development can be inherited by subsequent generations through IEI and TEI.

**Figure 3:**
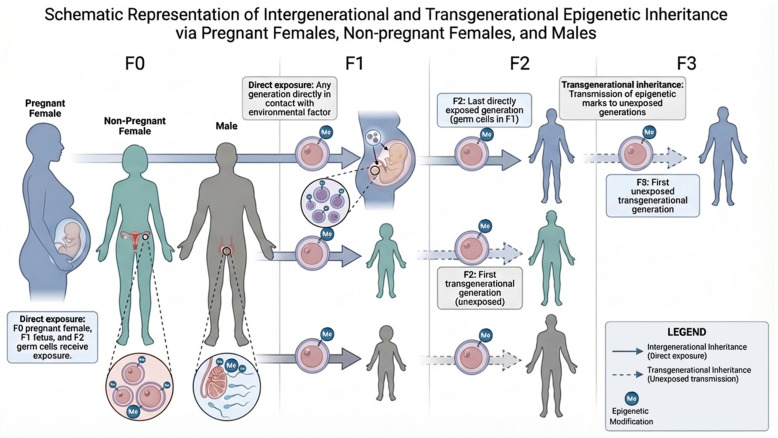
Intergenerational (IEI) and transgenerational epigenetic inheritance (TEI) across maternal and paternal exposure scenarios. Exposure of a pregnant female directly affects the mother (F0), the developing fetus (F1), and the germ cells within that fetus that will form F2, constituting IEI. Consequently, the first unexposed generation, and thus the first in which TEI can be assessed, is F3. In contrast, exposure of a non-pregnant female affects only the germ cells, producing IEI in F1; TEI can therefore first be evaluated in F2. Similarly, direct exposure of males affects their germ cells (F1), making F2 the first unexposed generation in which TEI may be observed.

During early embryonic development, germline-derived environmentally induced epigenetic alterations are challenged by the intrinsic and extrinsic factors that test their fidelity of inheritance to the next generations. Those that overcome this bottleneck are named as ‘escapee’ loci and demonstrate IEI or TEI across the following generations. IEI and TEI are long-recognized phenomena describing the inheritance of phenotypes across generations [2,8,42,61]. IEI captures those effects that are transmitted to the directly exposed offspring (F1) and, in the case of pregnant females, the germ cells of the F2 generation (Figure 3). On the other hand, TEI refers to phenotypic and molecular effects that persist beyond these directly exposed generations, implying stable propagation of epigenetic information through the germline (Figure 3) [11,42]. A study can be considered a strong candidate for environmentally induced IEI or TEI of neurodevelopmental and cognitive traits when it satisfies five key criteria: exposure to an environmental stimulus, evidence of an altered epigenetic mark, corresponding changes in gene expression, a measurable phenotypic effect, and the presence of germline epigenetic alterations in each generation [4,11]. For TEI, additionally, altered epigenetic marks and phenotypic differences should be transmitted to at least the first unexposed generation, as further illustrated in Figure 3. Table 1 summarizes the IEI and TEI studies that meet at least four of these criteria for environmentally induced epigenetic inheritance of neurodevelopmental and cognitive traits in mammals. Although the vast majority of studies in this area rely on rodent models, no direct studies of epigenetic inheritance for neurodevelopmental or cognitive traits have yet been conducted in sheep, despite their closer developmental alignment with humans.

Of the studies summarized in Table 1, six appear to meet the proposed criteria for IEI and five for TEI. An additional seven IEI and seven TEI studies fulfill most criteria but lacks either documented phenotypic differences or clear germ-cell epimutations. For instance, one TEI study showed that a maternal gestational high-fat diet induced insulin resistance and led to impaired synaptic plasticity, learning, and memory in unexposed F3 offspring, accompanied by persistent reductions in hippocampal *Bdnf* expression (F1–F3) and decreased germline H3K9ac/H3K4me3 at *Bdnf* promoters (F0) in mice [127]. Another study demonstrated that paternal preconception immune activation alters sperm small-RNA profiles, hippocampal gene expression, and behavior across exposed and unexposed generations, despite limited overlap in specific small-RNA species [129]. Although no direct relationship was observed between the differentially expressed sperm miRNA/piRNAs and the differentially expressed hippocampal genes, behavioral divergence was observed between the groups across generations, accompanied by miRNA and gene expression alterations [129]. This indicates that TEI may not require the persistence of identical epimutations across generations but instead reflects interactions among multiple epigenetic transmission mechanisms.

Beyond the studies summarized in Table 1, many reports suggest IEI or TEI but lack clear evidence linking environmental exposures to altered epigenetic marks and to a defined mechanistic pathway connecting these changes to the observed phenotypes [112,139,140,141,142,143]. Because altered epigenetic marks constitute the mechanistic bridge between environmental exposure, germline epimutations, their transmission across generations, and resulting traits, studies lacking this evidence cannot be interpreted as demonstrating IEI—and certainly not TEI—but instead point to exposure-related metabolic or associative effects. In addition, some studies use the terms IEI and TEI interchangeably, even when the evidence supports only IEI. For example, maternal stress during pregnancy was described as TEI, although the F2 generation remained directly exposed via in utero F1 germ cells [144]. Similarly, maternal immune activation was proposed to induce TEI of dopaminergic dysfunction, yet epigenetic differences were not detected beyond F1 despite phenotypic changes persisting to F3 [145]. Several additional studies report IEI of neurodevelopmental and cognitive traits but incorrectly label these effects as TEI [146,147].

Various studies summarized in Table 1 lacked phenotypic observations in the study populations; however, they identified differentially regulated and differentially methylated genes enriched for neurodevelopmental functions [120,121,132,133,134,135]. Most of these studies used environmental stimulants with widely recognized and well-established epigenetic regulatory functions such as folate and endocrine disruptors [121,132,133,134]. Folate, as a cofactor of one-carbon metabolism, provides single-carbon units for the synthesis of purines, pyrimidines (i.e., DNA/RNA nucleotide synthesis), regeneration of methionine, and damage repair mechanisms and methylation [22,148,149]. Most studies attribute the neurodevelopmental effects of folic acid to an interplay between folate and epigenetic regulation of gene expression, via one-carbon metabolism, which provides methyl groups to DNMTs for use across the genome [150,151,152]. Strikingly, another methyl donor of one-carbon metabolism, methionine, found by our studies to induce a five-generation TEI cascade, was characterized by differentially methylated genes enriched for neurodevelopmental processes when paternally supplemented through the diet of prepubertal sheep [3]. Although no neurological traits were observed in this study, differentially methylated genes in each generation and across unexposed generations were functionally annotated to regulate synaptic signaling, neuronal development, axon guidance, brain development and behavior [3].

## 6. Challenges for Epigenetic Studies of Neurodevelopmental and Cognitive Traits

As discussed in Section 3 and Section 4, crosstalk and information exchange among epigenetic molecular carriers in the germline, together with waves of epigenetic reprogramming compounded by zygotic genome activation and additional sources of variability in early embryos, represent major biological bottlenecks to obtaining empirical evidence for epigenetic transmission for neurodevelopmental and cognitive traits. Another major confounder is the absence of a unified conceptual definition of IEI and TEI, as reflected in the substantial variability among the studies reviewed in Section 5. Conceptually, the discipline lacks a pragmatic definition of TEI, leading to widespread confusion between intergenerational effects (Figure 3), correlational outcomes driven by other factors, and true germline-mediated inheritance that also manifests in unexposed descendants [2,4,11,42,153]. This conceptual ambiguity is further compounded by the complexity of mammalian reproduction, which introduces unavoidable confounding effects from the intrauterine environment and maternal physiology, as well as from lactation, the postnatal environment, parental behavior, and, particularly in human studies, cultural and ecological inheritance. These factors, independent of any germline epimutations, can alter the offspring’s epigenetic landscape through multidimensional environmental exposures, making it difficult to disentangle the specific contribution of the exposure of interest.

Another major challenge is disentangling environmentally induced inherited epimutations from those arising from DNA sequence variation [11,153]. Similar to environmental stimuli, genetic variation can also generate epimutations. This makes it difficult to distinguish genetic inheritance from genuine environmentally mediated epigenetic transmission and to determine their respective contributions to phenotypic traits, an issue that remains a widely criticized aspect of epigenetic inheritance research [2,11]. Additionally, to influence neurodevelopmental and cognitive functions, germline epimutations—beyond those already functional during early embryonic development—must persist throughout developmental trajectories and remain functionally relevant to nervous system and brain formation. Although relatively few, some studies discussed in Section 5 have demonstrated that inherited germline epimutations can propagate across relevant tissues. However, this requirement is complicated by extensive crosstalk and information exchange among epigenetic mechanisms during early embryogenesis. Such interactions may modify, overwrite, or reinterpret an initial germline epimutation while still producing similar phenotypic outcomes across generations [42,60,153,154]. This dynamic remodeling introduces an additional layer of mechanistic ambiguity, further complicating efforts to attribute a stable causally continuous epimutation to IEI or TEI.

The challenges become even more pronounced for the nervous system, neurodevelopmental, and cognitive phenotypes, which are highly polygenic, environmentally sensitive, and developmentally dynamic. Brain epigenetic states vary across cell types and developmental stages, and behavioral outcomes are strongly shaped by maternal care, stress, housing conditions, and social interactions. Combined with the high plasticity of the nervous system, these factors make it extremely difficult to attribute neurobehavioral traits specifically to germline-transmitted epigenetic marks rather than to developmental plasticity or cross-generational cultural and behavioral transmission [155]. On the other hand, establishing a causal link between epimutations and neurodevelopmental or cognitive traits is further complicated by the inherently complex and often highly qualitative nature of these phenotypes. Disorders such as ASD and ADHD are defined by broad heterogeneous criteria that rely heavily on categorical clinician-dependent assessments rather than continuous biomarker-anchored measurements [156]. When traits lack quantitative scales or objective biological readouts, their evaluation becomes intrinsically subjective, making it substantially more difficult to trace the inheritance of environmentally induced epimutations or to attribute phenotypic variation to specific epigenetic mechanisms across generations. Finally, data interpretation is hindered by small effect sizes, tissue heterogeneity, batch effects, and multiple-testing burdens, all of which increase the risk of false positives. Consequently, the reproducibility of epigenetic association across laboratories, strains, and species remains inconsistent and often questionable [4].

Most evidence for epigenetic inheritance in mammals is associative: environmental exposures, altered epigenetic marks in sperm or oocytes, and offspring phenotypes often coincide, but clear causal chains are rarely established, particularly for neurodevelopmental and cognitive traits. Germline and early embryonic reprogramming waves should, in theory, erase most acquired marks. Demonstrating that specific germline epimutations escape these waves and remain functional in neural lineages is still rare and technically challenging. An integrated evidence chain, from a germline epimutation to an early embryonic signature, an altered neurodevelopmental program, a defined circuit or synaptic phenotype, and ultimately a cognitive outcome, has yet to be established. Most studies stop at one or two of these levels.

## 7. Future Directions

Future research on the epigenetic studies of neurodevelopmental and cognitive traits will require a coordinated frameshift toward more robust experimental design for causal inference, mechanistic precision, and phenotypic definitions. A critical priority is the establishment of unified pragmatic definitions that clearly delineate intergenerational from transgenerational effects, specify what constitutes an epimutation underlying a trait, and articulate the developmental windows during which such carriers can plausibly influence neural lineage specification. To overcome some of the challenges and confounders covered earlier, several studies suggested roadmaps and criteria for pragmatic and empirically sound epigenetic inheritance studies, especially for TEI [4,11,153]. For IEI and TEI of neurodevelopmental and cognitive traits, progress will depend on integrated approaches. These include multi-omics profiling of germline and early embryos (genome, epigenome, transcriptome, proteome, and metabolome). They also require dense and precise measurements of environmental, behavioral, and physiological factors across generations. In parallel, affected tissues must be profiled. Together, these approaches will allow experimental models to disentangle genetic, epigenetic, and purely environmental transmission pathways. For cognitive outcomes, further progress requires moving beyond broad categorical psychiatric diagnoses toward quantitative biomarker-anchored phenotypes. Approaches such as neuroimaging, electrophysiology, digital behavioral assays, and dimensional cognitive measures can help reduce subjectivity and the margin of error in disease diagnosis.

We argue that the epigenetic regulation of phenotypic outcomes, particularly when inheritance is considered, should no longer be viewed as a single-layer mechanism. Instead, multiple epigenetic processes and their multilayer interactions contribute to the emergence of observable phenotypes, as highlighted throughout several sections and in Table 1. This complexity emphasizes that trait expression reflects the integrated output of intersecting epigenetic processes rather than the action of any isolated mechanism. Therefore, future studies interrogating epigenetics of neurodevelopmental and cognitive traits require multiple layers of epigenetic markers to be screened within the same experimental population, through a holistic approach that incorporates whole-genome DNA methylation, histone modifications, 3D genome organization, non-coding RNAs, and mRNA methylation. Furthermore, we suggest that these layers be incorporated into high-resolution epigenome profiling (e.g., WGBS, ATAC-seq, ChIP-seq, CUT&RUN/CUT&Tag, Hi-C sequencing, small RNA sequencing, and MeRIP-seq/m6A-seq technologies) across both germline cells and affected tissues. This is also expected to facilitate capturing the crosstalk among the epigenetic mechanisms regulating the gene expression patterns throughout the inheritance of these complex traits [153]. It is also important to note that long-read sequencing approaches currently offer higher precision and accuracy for low input germline and embryo epigenome profiling.

To further elucidate the causality and persistence of environmentally induced epimutations, we anticipate that recent advances in single-cell technologies will be transformative. These approaches can now be coupled with multi-omics profiling across the earliest stages of development, capturing DNA methylation, 3D genome organization, histone modifications, small RNAs, and transcriptional states within the same cell or lineage trajectory [157]. When combined with multi-tissue and multi-layer high-resolution epigenome profiling during periods of trait expression, these integrative frameworks will allow researchers to trace how environmentally induced epimutations emerge, propagate, and are remodeled across developmental time. This level of resolution is essential for determining whether specific epimutations persist through developmental trajectories and exert meaningful influence on neurodevelopmental and cognitive phenotypes across generations. Furthermore, epigenome editing platforms allow researchers to experimentally recreate, erase, or invert putative environmentally induced epimutations in germ cells, early embryos, or relevant neural lineages. Targeted alterations offer a robust way to determine whether a specific epigenetic modification is sufficient or necessary to influence gene regulation, development, or neurobehavioral phenotypes. As CRISPR-based epigenome editing tools gain precision, efficiency, and cell-type specificity, they will become essential for establishing mechanistic causality of epimutations, particularly for complex neurodevelopmental and cognitive traits where observational evidence alone is inadequate.

From a translational perspective for humans, selecting an appropriate animal model is crucial for advancing epigenetic research in neurodevelopmental and cognitive traits. Most studies examining environmental exposures and epigenetic alterations affecting nervous system development and cognition have relied on mice or rats (Table 1). Although rodent models have provided valuable insights, their relevance to human neuroscience is limited, as discussed previously [14,25,158,159]. Emerging evidence suggests that small rodents may not adequately model key aspects of human neurodevelopmental disorders, underscoring the need to incorporate large-animal systems. We therefore propose that species such as sheep—whose gestational biology, brain development, and perinatal physiology more closely resemble those of humans—can serve as valuable models for investigating the phenotypic consequences of epigenetic inheritance in a translationally relevant context. Additional approaches to enhance translational relevance include studying epigenetic mechanisms of neurodevelopment directly in human cells using induced pluripotent stem cells and advanced 3D cerebral organoids [157].

Together, these approaches will establish the conceptual and methodological framework needed to determine whether environmentally induced epimutations can meaningfully influence neurodevelopment and cognition across generations and to define the biological constraints under which such inheritance occurs.

## 8. Conclusions

This review integrates evidence from germline epigenetics, early embryonic patterning, neurodevelopment, and epigenetic inheritance to propose a unified mechanistic framework linking environmentally induced epimutations to neurodevelopmental and cognitive outcomes. By integrating the findings across these traditionally compartmentalized fields, we highlight how environmental signals encountered by gametes and early embryos can reshape the epigenetic landscape during periods of profound developmental plasticity, influence lineage allocation during gastrulation, and ultimately alter the molecular architecture of the developing central nervous system. The sensitivity of neurodevelopmental trajectories to early epigenomic instability also highlights the plausibility of both IEI and TEI of neurodevelopmental and cognitive traits. At the same time, our synthesis reveals substantial gaps in the current evidence base, including the limited number of studies that meet all criteria required to establish a strong case for epigenetic inheritance of these phenotypes. We also emphasize the need for more translationally relevant models, as developmental and epigenetic timing in rodents diverges markedly from that in humans, whereas large-animal systems such as sheep offer closer alignment during the earliest stages of embryogenesis. Together, these insights argue for a systems–biology approach that spans germline biology, embryonic epigenetic reprogramming, and neural development to fully understand how early exposures generate long-lasting and, in some cases, heritable effects on CNS development and function. Advancing this integrative framework will be essential for clarifying causal mechanisms, improving model selection, and ultimately informing strategies to mitigate environmentally driven neurodevelopmental risk.

## Figures and Tables

**Table 1:** Intergenerational (IEI) and transgenerational epigenetic inheritance (TEI) studies of environmentally induced epigenetic changes in neurodevelopmental and cognitive traits in mammals.

Environmental Stimuli	Species	Epigenetic Mark	Inheritance	Phenotype	Unobserved Criteria	Study
Paternal advanced age	Mice	Sperm DNA methylation	IEI	Learning and memory	-	[114]
Paternal 5G RFR exposure	Mice	Sperm DNA methylation	IEI	Anxiety-like behavior and impaired sperm quality in F1 males	-	[115]
Paternal preconception methyl donor-rich diet	Mice	Sperm and hippocampus DNA methylation	IEI	Deficits in hippocampus-dependent learning and memory, impaired hippocampal synaptic plasticity, and reduced hippocampal theta oscillations	-	[116]
HFD-induced preconception paternal obesity	Mice	Sperm DNA methylation	IEI	Hippocampal neurogenesis and cognitive impairments in F1	-	[117]
Maternal alcohol consumption during pregnancy	Mice	Histone methylation, histone acetylation	IEI	Fetal alcohol syndrome-associated congenital abnormalities	-	[118]
Maternal hyperglycemia exposure during pregnancy	Mice	Sperm DNA methylation	IEI	Male-specific memory impairment	-	[119]
Male lifetime folic acid deficiency/Excess folic acid supplementation	Mice	Sperm, placenta, cortex imprinted genes’ DNA methylation	IEI	Increased offspring mortality	Phenotypic measurement	[120]
Maternal endocrine disruptor DDE exposure during pregnancy	Human	Sperm DNA methylation	IEI	Enrichment of genes associated with ASD and neurodevelopment	Phenotypic measurement	[121]
DEHP-administered pregnant females	Rat	Brain DNA methylation	IEI	Anxiety-like behavior, working memory impairment	Germ-cell epimutation	[122]
Maternal bisphenol A exposure during pregnancy	Mice	Hippocampus DNA methylation	IEI	Impaired hippocampus neurogenesis, neurocognitive deficit of memory retention	Germ-cell epimutation	[123]
Combined parental preconception spatial training	Rat	Hippocampus histone acetylation	IEI	Sex-specific induced spatial learning and memory improvement	Germ-cell epimutation	[124]
Maternal nicotine exposure during pregnancy	Mice	Striatum and frontal cortex DNA methylation	IEI	Enhanced nicotine preference, increased hyperactivity and risk-taking behaviors, perturbed rhythmicity of activity, ADHD-like symptoms	Germ-cell epimutation	[125]
Paternal early life protein-energy deficient malnutrition	Human	Blood DNA methylation	IEI	Impairments in attention and cognition, ADHD and IQ association	Germ-cell epimutation	[126]
Maternal high-fat diet induced insulin resistance during pregnancy	Mice	Sperm/hippocampus histone acetylation and methylation	TEI	Impaired synaptic plasticity, learning and memory	-	[127]
Maternal alcohol consumption during pregnancy	Rat	Sperm, hippocampus POMC DNA methylation, histone modifications	TEI	Male-specific stress-axis abnormalities, abnormal POMC neuronal functions	-	[128]
Paternal preconceptional immune activation by Poly I:C	Mice	Sperm miRNA + piRNA	TEI	Depression-like behavior, hippocampus transcriptome, immune responsivity	-	[129]
Maternal sevoflurane (general anesthesia) exposure during pregnancy	Mice	Sperm chromatin accessibility	TEI	ASD-like phenotypic observations, impaired sociability	-	[130]
Paternal stress through corticosterone exposure	Mice	Sperm miRNA only in F0	TEI	Hyper anxiety-like behavior in F1, lower levels of anxiety in F2, depression-like behavior in F2	-	[131]
Paternal endocrine disruptor exposure	Rat	Sperm and hippocampus DNA methylation	TEI	Enrichment of genes associated with nervous system development	Phenotypic measurement	[132]
Preconception and in utero maternal exposure groups: persistent organic pollutants (POPs), folate, POPs + folate	Rat	Sperm miRNA	TEI	Enrichment of genes in brain development	Phenotypic measurement	[133]
Paternal lifetime exposure to folate deficiency and excessive supplementation	Mice	Sperm DNA methylation	TEI	Enrichment of genes in nervous system development and neuronal action potential, particularly enriched for LINEs	Phenotypic measurement	[134]
Maternal stress exposure during pregnancy	Mice	Fetal cortex and placenta DNA methylation + miRNA profiles	TEI	Enrichment of genes associated with neurological and psychiatric diseases	Phenotypic measurement	[135]
Paternal nicotine exposure	Mice	Hippocampus DNA methylation	TEI	Enhanced fear memory formation and spontaneous recovery of fear memories	Germ-cell epimutation	[136]
Maternal sub-chronic inflammation caused by lipopolysaccharide (LPS) during pregnancy	Mice	Hippocampus histone methylation and acetylation	TEI	Spatial learning and memory impairment	Germ-cell epimutation	[137]
Paternal arsenic exposure	Rat	Hippocampus Histone modifications	TEI	Learning, memory, cognitive impairment	Germ-cell epimutation	[138]

## Data Availability

Data sharing is not applicable. No new data were created or analyzed in this study.

## Abbreviations

DOHaD	Developmental Origins of Health and Disease
IEI	Intergenerational Epigenetic Inheritance
TEI	Transgenerational Epigenetic Inheritance
PGC	Primordial Germ Cell
CNS	Central Nervous System
DNMT	DNA Methyltransferase
TET	Ten-eleven Translocation Protein
HDAC	Histone Deacetyltransferase
TAD	Topologically Associating Domain
SAM	Sadenosylmethionine
SAH	Sadenosylhomocysteine
NTD	Neural Tube Defect
TE	Transposable Element
ASD	Autism Spectrum Disorder
ADHD	Attention-Deficit/Hyperactivity Disorder
PCP	Planar Cell Polarity
